# Uncovering mechanisms in video game research: suggestions from the expert-performance approach

**DOI:** 10.3389/fpsyg.2014.00161

**Published:** 2014-03-03

**Authors:** Tyler J. Towne, K. Anders Ericsson, Anna M. Sumner

**Affiliations:** K. Anders Ericsson Laboratory, Department of Psychology, Florida State UniversityTallahassee, FL, USA

**Keywords:** expert performance, video games, deliberate practice, skill development, transfer of training

In the United States, video game playing is an immensely popular form of entertainment; the majority of adults will have had some experience with video games during their lives (Rideout et al., 2010). As the popularity of video games in entertainment has increased, so has interest in exploring the potential effects that video games may have on learning and generalizable cognitive ability. Educators have begun to seek a way to use video games as a tool to motivate students to learn academic skills (Blumberg, 2014). Despite general enthusiasm for video games as an avenue for training, there are shortcomings in the methodology commonly used to demonstrate the advantages of video game experience (See Boot et al., 2011; Kristjánsson, 2013). Additionally, few studies have sought to evaluate video game skill in the context of established research on skill acquisition in more traditional domains. This paper attempts to connect research on expertise with the claims being made in video game studies. Particularly, we discuss how the expert-performance approach can be used to describe video game performance and the mechanisms that are responsible for increases in skill as well as for potential transfer. We will also discuss how the design of traditional “casual” video games may be inconsistent with principles of skill acquisition through deliberate practice that has been documented in many other domains (Ericsson et al., 1993; Ericsson, 2006a).

## Using the expert performance approach to evaluate skill and transfer in video games

The expert-performance approach necessitates that researchers proceed through a series of steps. The first step requires demonstrating that some individuals are able to perform on a reproducibly superior level than others, when presented with a standard domain-representative task. For example, studies have demonstrated that after video game training, typically lasting 10–40 h, non-gamers show reliable individual differences in performance when they play the game “Space Fortress” (Green and Bavelier, 2006; Basak et al., 2008). One problem with analyzing these individual differences in total score on the game is that, based on the selection of the first few actions, participants will encounter very different problem spaces and thus their outcome scores are not comparable. For example, the differences in total score could depend on differences in strategies, speed and accuracy, perceptual-motor implementations, or other general abilities.

The methodology of the expert-performance approach remedies this issue by identifying a number of situations from a game environment where one would present participants with the task of executing an immediate short sequence of actions. This method (Ericsson and Smith, 1991; Ericsson, 2006b) was derived from de Groot's (1978) work in chess where he presented players with challenging chess positions and required that they select the best subsequent move. By using a standard representative task, it becomes possible to compare individuals of different skill levels within a narrowly defined problem space. Additionally, by limiting analyses to a small subset of these representative tasks, it becomes theoretically easier to understand the structure and underlying mechanisms supporting subjects' performance. This approach has been used to describe mechanisms of skill in soccer (Ward et al., 2013), snooker (Abernethy et al., 1994), SCRABBLE (Tuffiash et al., 2007), and typing (Keith and Ericsson, 2007), among others. Video game environments can be limited in such a way that small snapshots of video game performance can be isolated from the larger game in order to present players with consistent scenarios such that differences between more and less skilled subjects can be identified.

Once performance on a set of representative tasks has been measured, the expert-performance approach attempts to trace the processes mediating the superior performance. The most influential method involves collecting concurrent and retrospective verbal report data (Ericsson, 2006b) from participants during performance on representative tasks. The methods of concurrent and retrospective verbalization draws on fundamentally different cognitive processes for their generation than the less successful traditional interviews with experts to extract rules for expert systems (Ericsson and Simon, 1993; Fox et al., 2011). For example, Moxley et al. (2012) analyzed think aloud protocols of chess players selecting moves to assess intuition's role in generating superior moves by highly rated players. There has also been studies collecting think aloud verbalizations while playing entire games (Blumberg et al., 2008; Blumberg and Randall, 2013).

Significant work has been done designing experimental manipulations that interfere with task performance to test hypotheses about the mediating processes revealed by verbal reports. For example, individuals with exceptional memory have had their memory performance reduced to the level of college students by manipulating the material and conditions of memorization (Ericsson and Polson, 1988; Ericsson et al., 2004; Hu et al., 2009; Hu and Ericsson, 2012). Based on verbal reports collected from representative situations in particular video games, it should be possible to generate experimental manipulations that would interfere with the processes reported by systematically changing the game environment. Finally, one would attempt to trace the development and acquisition of the various mechanisms that are found to mediate the superior performance and assess the role of prior deliberate practice and innate talents in their development.

By using the expert-performance approach to systematically evaluate the nature of skill in video games, researchers can begin to make more specific hypotheses about the mechanisms that account for findings of generalizable transfer. By using this approach in other domains, studies have found that skills previously explained by generalizable mechanisms are instead the result of the accumulation of highly domain-specific cognitive structures (Ericsson et al., 1980, 2004; Ericsson and Kintsch, 1995). Additionally, the expert-performance approach will allow video game researchers to objectively quantify skill by implementing the use of standard representative tasks, where traditionally, they have relied on a gamer vs. non-gamer distinction (cross-sectional) or total game score (longitudinal) when making claims about the cognitive advantages of skilled video game players.

## Video games as training tools

Studying video game performance is particularly appealing to researchers because, traditionally, games have been explicitly designed to keep players' attention, maintain an enjoyable level of challenge, and lead to continued improvements across many hours of play to give a sense of accomplishment. We are particularly interested in contrasting skill acquisition in popular video games to more traditional domains where effective practice activities have been identified. Studies have discovered a pattern of behaviors, known as deliberate practice, in areas such as music, chess, and sport that are highly predictive of skilled performance (Ericsson et al., 1993). Deliberate practice is defined as the engagement, with full concentration, in a training activity designed to improve a particular aspect of performance with immediate feedback, opportunities for gradual refinement by repetition, and problem solving.

**Figure 1:**
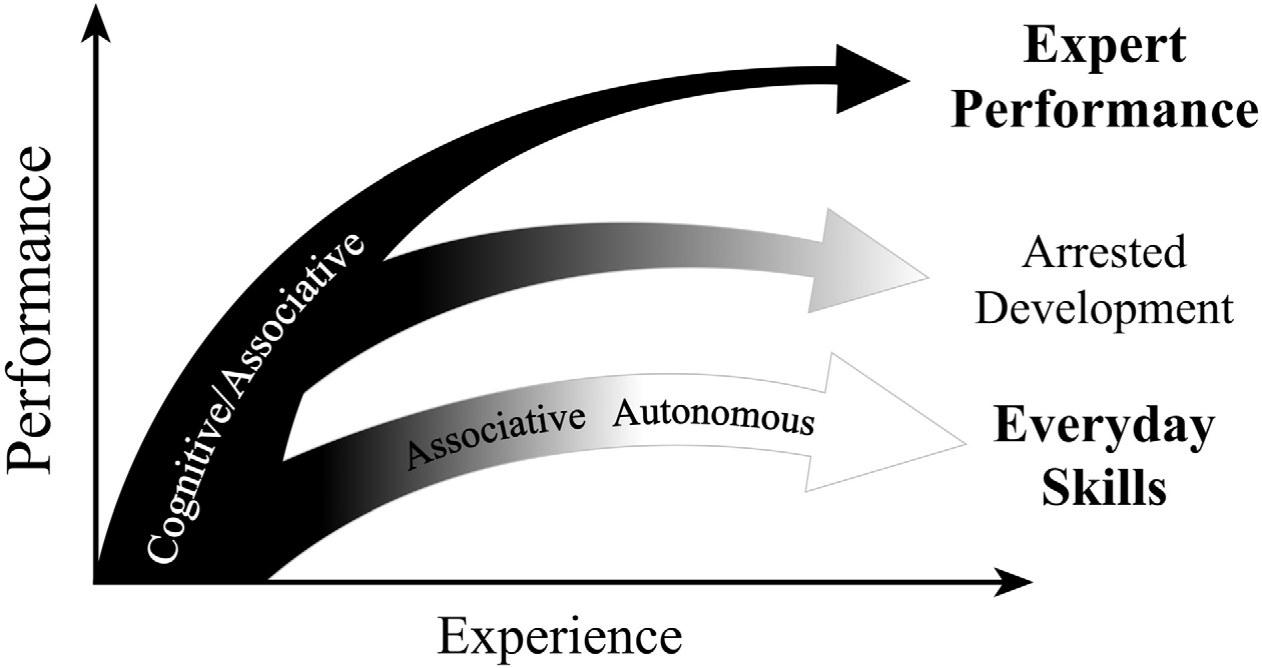
**An illustration of the qualitative difference between improvement of experts and those engaging in a domain recreationally**. The goal for casual players is to quickly reach a satisfactory level that is stable and “autonomous,” at which point, positive feedback is a much more common than negative feedback. In contrast, expert performers counteract automaticity by developing increasingly complex mental representations to attain higher levels of control of their performance. Therefore, they remain in the “cognitive” and “associative” phases. Some experts will, at some point in their career, stop engaging in deliberate practice and prematurely automate their performance. (Adapted from “The scientific study of expert levels of performance: General implications for optimal learning and creativity” by K. A. Ericsson in High Ability Studies, 9, p. 90. Copyright 1998 by European Council for High Ability).

Effective practice activities should be designed to foster continued development. In areas such as driving, typing, and recreational sports, an adequate level of performance is reached, productions are automated, appropriately challenging situations are not sought, and improvement is arrested (see Figure 1). In competitive games, such as baseball, chess, and soccer, the level of challenge typically increases as the participating individuals improve their skill. One of the key characteristics of traditional video games is that the difficulty level of a game is adjusted as the player masters a given level and progresses to the next. This adaptable level of difficulty gives the player an appropriate sense of challenge and interest and the change in difficulty is often associated with the introduction of new and unfamiliar environments.

Many solitary videogames will allow the player to pursue a path until they are not able to handle a situation, at which point they are reset to the beginning of the current level. In the past, this meant that players would spend much of their time re-tracing steps until returning back to the challenging situation, an act unrelated to skill acquisition. More recent games have built in mechanisms to “save” a game before reaching a challenging point so that the mastered parts of a level would not need to be retraced. It would therefore be interesting to analyze the amount of time that a player engages in activities successfully (positive feedback), as well as how often they fail (negative feedback), and the associated competitive outcomes.

Ericsson et al. (1993) found that individuals enjoyed successfully playing music, soccer and baseball games, and chess matches. However, the time spent in these types of successful performances were not associated with engagement in activities that are designed to maximize learning. To apply what we know about deliberate practice to video games, one would have to look for specific challenges encountered in games where players have the option to replay these situations either through a “rewind” or “save” mechanism. Players would then be able to replay the challenge repeatedly until they feel they had mastered the situation.

When Ericsson et al. (1993) studied how highly skilled individuals spent their time improving their skills, they engaged in exactly this type of deliberate practice. A music student encountering a problem with one section of a piece of music would not simply rehearse the piece again and again, he or she would focus on the difficult part and repeatedly work on mastering just that section before returning to the entire piece. Similarly, skilled chess players study positions from games of chess masters to find the best move. Once they have generated their best move for the position they can compare their move to the chess master's selected move during that game. This gives immediate feedback instead of completing a chess game across several hours and then trying to identify where they could have selected a better move.

Deliberate practice requires that individuals engage in training at the limits of their ability, where they often fail. It is not as enjoyable as tasks, like play, where performance can be generated easily and effortlessly. One method to make the activity attractive is to mix a short duration of deliberate practice (10–15 min for beginners) with the majority of time being spent on play. Many video games are structured in a similar manner by having the player spend most time on already mastered activities until they reach challenges, but after a few they are sent back to familiar territory.

Video games offer researchers an opportunity to study skill development in a relatively well-controlled environment, where they can manipulate specific parameters of the game. It is relatively easy to maintain a minimum level of experimental control and log data in such a way that specific behaviors can be isolated and related to subsequent performance gains. However, it is difficult to draw generalizable conclusions about the nature of skill development when examining games in which positive feedback outweigh the instances of negative (i.e., casual games).

## Conclusions

We believe that applying the expert-performance approach to skill in video game environments is essential for understanding the mechanisms of superior performance as well as the shared components that may eventually account for discovered correlations between video games and general ability measures. Furthermore, we believe that it is unlikely that classic game environments are optimally designed to foster continued improvement in an ecologically valid way.

Many of the video games that have been proposed in the literature as vehicles for academic and real-world improvement are those such as Space Fortress, Medal of Honor, Rise of Nations, and Tetris that seem to be highly self-motivating, primarily because they give more positive feedback than informative negative feedback. We argue that meaningful improvement is only achieved through principles of deliberate practice, which include negative feedback (i.e., failures), and that many traditionally studied games do not adequately incorporate these components. Superior skill is the product of many years and decades of intense training under conditions that are very different from those in a typical video game. If meaningful real-world tasks were designed like a casual or recreational video game environment, it is unlikely that individuals would improve beyond a relatively low and automated state, which is the observed outcome of extended experience in most professional environments (Ericsson, 2006a). As with any proposed training regimen, video game training must be systematically evaluated against alternatives. Our assertion is that the very components of video games that make them uniquely motivating, are antithetical to the deliberate practice. Additionally, only through systematic detailed descriptions of individuals' behavior and acquired skills can we begin to hypothesize about the role that acquired performance in video games might benefit cognitive development in schools and everyday life.
